# Resveratrol Promotes Angiogenesis in a FoxO1-Dependent Manner in Hind Limb Ischemia in Mice

**DOI:** 10.3390/molecules26247528

**Published:** 2021-12-12

**Authors:** Dongxiao Fan, Chenshu Liu, Zeling Guo, Kan Huang, Meixiu Peng, Na Li, Hengli Luo, Tengyao Wang, Zhipeng Cen, Weikang Cai, Lei Gu, Sifan Chen, Zilun Li

**Affiliations:** 1 Division of Vascular Surgery, The First Affiliated Hospital of Sun Yat-sen University, Guangzhou 510080, China; fandx13@163.com (D.F.); oscar_liu1995@outlook.com (C.L.); huangkan1986@hotmail.com (K.H.); husterlina@163.com (N.L.); 2 National-Guangdong Joint Engineering Laboratory for Diagnosis and Treatment of Vascular Diseases, The First Affiliated Hospital of Sun Yat-sen University, Guangzhou 510080, China; meix1124@163.com; 3 Department of Otolaryngology, The First Affiliated Hospital of Sun Yat-sen University, Guangzhou 510080, China; guozling@mail2.sysu.edu.cn; 4 Guangdong Provincial Key Laboratory of Malignant Tumor Epigenetics and Gene Regulation, Guangdong-Hong Kong Joint Laboratory for RNA Medicine, Sun Yat-Sen Memorial Hospital, Sun Yat-Sen University, Guangzhou 510120, China; luohli@vip.163.com (H.L.); wangty66@mail2.sysu.edu.cn (T.W.); cenzhp@mail2.sysu.edu.cn (Z.C.); 5 Medical Research Center, Sun Yat-Sen Memorial Hospital, Sun Yat-Sen University, Guangzhou 510120, China; 6 Department of Biomedical Sciences, New York Institute of Technology, College of Osteopathic Medicine, Old Westbury, NY 11568, USA; Wcai04@nyit.edu; 7 Max Planck Institute for Heart and Lung Research and Cardiopulmonary Institute (CPI), 61231 Bad Nauheim, Germany; Lei.Gu@mpi-bn.mpg.de

**Keywords:** hind limb ischemia, therapeutic angiogenesis, resveratrol, FoxO1, metabolomics

## Abstract

Critical limb ischemia (CLI) is a severe form of peripheral artery diseases (PAD) and seriously endangers the health of people. Therapeutic angiogenesis represents an important treatment strategy for CLI; various methods have been applied to enhance collateral circulation. However, the current development drug therapy to promote angiogenesis is limited. Resveratrol (RSV), a polyphenol compound extracted from plants, has various properties such as anti-oxidative, anti-inflammatory and anti-cancer effects. Whether RSV exerts protective effects on CLI remains elusive. In the current study, we demonstrated that oral intake of RSV significantly improved hind limb ischemia in mice, and increased the expression of phosphorylated Forkhead box class-O1 (FoxO1). RSV treatment in human umbilical vein endothelial cells (HUVECs) could increase the phosphorylation of FoxO1 and its cytoplasmic re-localization to promote angiogenesis. Then we manipulated FoxO1 in HUVECs to further verify that the effect of RSV on angiogenesis is in a FoxO1-dependent manner. Furthermore, we performed metabolomics to screen the metabolic pathways altered upon RSV intervention. We found that the pathways of pyrimidine metabolism, purine metabolism, as well as alanine, aspartate and glutamate metabolism, were highly correlated with the beneficial effects of RSV on the ischemic muscle. This study provides a novel direction for the medical therapy to CLI.

## 1. Introduction

With high prevalence of diabetes and obesity, as well as the rise of aging population, peripheral artery disease (PAD) has become a global health issue affecting more than 200 million persons [1,2]. Critical limb ischemia (CLI) is the most severe form of PAD. It has been reported that 20% of patients with chronic CLI would die within the first year of symptom onset [3]. Wound, ischemia and foot infection are three major factors contributing to threatened lower limb (TLL), and ischemia is the dominant risk for amputation [4]. For the treatment of PAD, revascularization is an effective therapy. However, there are several limitations for the application of revascularization: (1) the optimal timing and indications remain controversial; (2) some patients may suffer from severe postoperative complications; and (3) some patients might not be suitable for revascularization [5]. Hence, pharmacotherapy is still widely used with a considerable treatment efficiency. Contemporary drugs for PAD are mainly used for treating atherosclerosis to alleviate symptoms and to reduce cardiovascular events; however, the medicine for severe forms of PAD such as CLI is scarce.

Medical management for improving angiogenesis remains a promising research field [6]. Since the concept of therapeutic angiogenesis was initiated in 1994 [7], various treatments have been applied for ischemic diseases. Initially, vascular endothelial growth factor (VEGF), one of the proangiogenic cytokines, was administered as an intra-arterial bolus for the treatment of rabbit hind limb ischemia [7]. Although preliminary clinical trials showed that VEGF treatment of PAD was safe, the therapy efficacy remained controversial [8]. In recent years, stem cell transplantation [9] and exosome intervention [10] have been recognized as new treatment strategies to induce angiogenesis. Nakagami et al. transplanted autologous adipose-derived stem cells (ADSC) in a mouse hind limb ischemic model and found that ADSC could favor angiogenesis in ischemic skeletal muscle tissue [11]. Takerra et al. demonstrated that exosomes secreted by vascular progenitor cells can stimulate angiogenesis in vivo and in vitro [12]. For the same purpose, small molecule compounds have also been widely adopted to treat ischemic diseases because of their inherent advantages, such as non-invasion, long lasting and stable concentration in the body. Yin et al. demonstrated that Danshensu could accelerate angiogenesis in rat myocardial infarction model [13]. Zhang et al. found the role of Baicalin in angiogenesis by increasing VEGF in different cell lines [14].

Resveratrol (RSV) has been widely studied as an anti-inflammatory, anti-oxidative, anti-platelet and anti-aging compound [15]. However, the explicit effect of RSV on angiogenesis remains controversial. Huang et al. demonstrated that RSV could promote diabetic wound healing by activating the pathway related to angiogenesis [16]. Rong et al. found that RSV promoted angiogenesis by decreasing the expression of VEGF and Ang II and increasing Ang I in severe acute pancreatitis patients [17]. On the contrary, in different rodent cancer models, RSV has demonstrated the ability to reduce apoptosis and angiogenesis to inhibit the initiation and growth of tumors [18]. Bhatt et al. used RSV as a drug to inhibit angiogenesis in age-related macular degeneration in in vitro experiment [19]. In summary, the role of RSV on angiogenesis is unclear, and the dependent pathway remains elusive. Hence, we aim to test the exact effect of RSV on CLI and to clarify the underlying mechanism.

In this study, we explored the protective effects of RSV on hind limb ischemia mice by observing blood flow recovery after intervention. Then we identified the pathway in vivo and in vitro and manipulated human umbilical vein endothelial cells (HUVECs) to verify the pathway dependent manner after treatment with RSV under hypoxia condition. Finally, we performed metabolomics study to discover the metabolic changes in all experimental groups, and enriched the pathway to clarify the mechanism of the beneficial role of RSV for CLI. Our research suggests a new direction for the RSV treatment for CLI.

## 2. Results

### 2.1. RSV Treatment Improves Blood Restoration in Hind Limb Ischemia Mouse Model

**Figure 1:**
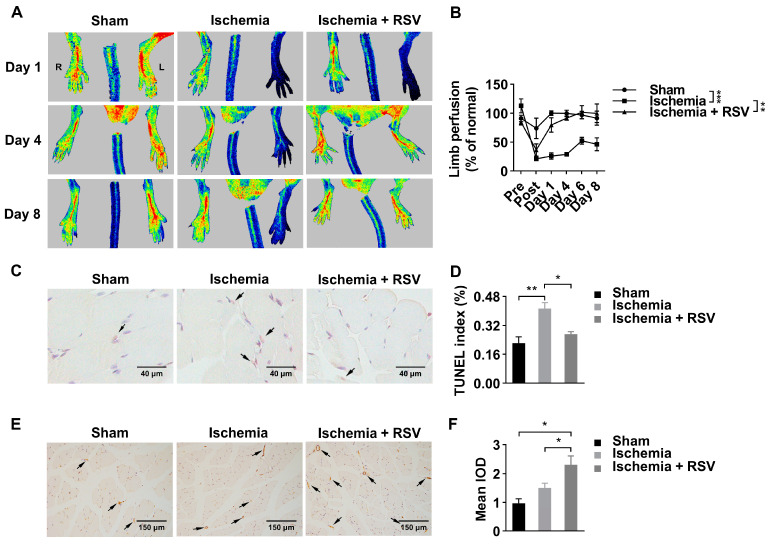
RSV promoted blood flow recovery and neovascularization in ischemic hind limbs of mice. (**A**) Laser Doppler measurement of hind limb blood flow was assessed 1 day before ischemic surgery as well as 0, 1, 4, 6, and 8 days after surgery. Red, green and blue represent high, medium and low blood perfusion degree, respectively. (**B**) Quantitative analysis of the blood flow perfusion ratio of ischemic-to-non-ischemic hind limbs. Data were shown as mean ± SEM (*n* = 3–4, ** *p* < 0.01; *** *p* < 0.001). Gastrocnemius tissues were collected and processed for TUNEL staining (**C**) (400×, scale bar = 40 μm) and CD31 staining (**E**) (200×, scale bar = 150 μm). Quantitative analysis of the three groups (**D**, **F**) were evaluated. Data were shown as mean ± SEM (*n* = 3, * *p* < 0.05; ** *p* < 0.01). Ischemia: mice performed with femoral artery severance surgery; Ischemia + RSV: hind limb ischemia mice administrated with resveratrol; Sham: mice performed with temporal femoral artery ligation for 5 min. IOD: integral optical density.

To determine the effects of RSV on ischemic mice hind limbs, RSV was administrated orally. The blood perfusion was measured by a laser Doppler imager (Figure 1A). As shown in Figure 1A,B, after hindlimb ischemia surgery, the blood flow perfusion was reduced significantly, while no significant changes of blood flow perfusion were observed in ischemic limbs in the sham group. At day 4 after surgery, the blood flow perfusion of ischemic hind limbs in the ischemia + RSV group showed a full recovery to the pre-surgery level, whereas the ischemia group showed a recovery of 25.62% to the pre-surgery level. Apoptosis was assessed by Terminal-deoxynucleotidyl transferase mediated nick end labeling (TUNEL) staining (Figure 1C). The TUNEL staining suggested that apoptotic cells in gastrocnemius muscles were significantly increased in the ischemia group, and decreased to a normal level in the ischemia + RSV group when compared to the sham group (Figure 1D). To determine the biomarker of angiogenesis in gastrocnemius muscles, immunohistochemical staining was performed to detect the expression level of CD31 (Figure 1E). The result indicated that the vessel density was increased in the ischemia group and further increased in the ischemia + RSV group (Figure 1F). These findings suggested that RSV possessed significant protective effect against hind limb ischemia.

### 2.2. RSV Inhibits FoxO1 Pathway in Ischemic Muscle and HUVECs

**Figure 2:**
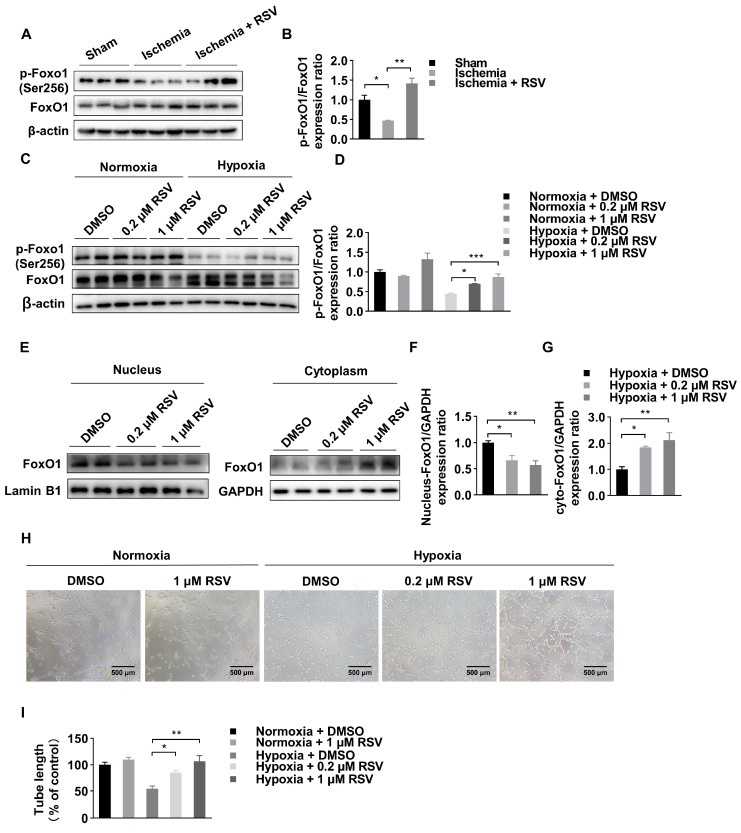
Identification of FoxO1 change in vitro and in vivo. (**A**) Ischemic skeletal muscles lysates were used to detect the FoxO1 and the phosphorylation of FoxO1 by Western blot. (**B**) Quantitative analysis of Western blot. Data were shown as mean ± SEM (*n* = 3, * *p* < 0.05; ** *p* < 0.01). (**C**) Total expression of FoxO1 and phosphorylation of FoxO1 under normoxia and hypoxia conditions in vitro. HUVECs were treated with DMSO or RSV for 0.2 and 1 μM for 6 h. (**D**) Quantitative analysis of Western blot. Data were shown as mean ± SEM (* *p* < 0.05; *** *p* < 0.001). (**E**) RSV treatment led to nuclear-cytoplasmic shuttling in HUVECs as determined by Western blot of nuclear and cytoplasmic lysates, cells were treated with DMSO or RSV for 0.2 and 1 μM for 6 h under hypoxia condition. (**F**,**G**) Quantitative analysis of Western blot. Data were shown as mean ± SEM (* *p* < 0.05; ** *p* < 0.01). (**H**) Hypoxic pretreatment and normal HUVECs were seeded in 96-well plates precoating Matrigel, and incubated with RSV of 0.2 and 1 μM for 8 h under hypoxia condition. Capillary like tube formation was captured by a digital camera attached to a microscope (100×, scale bar = 500 μm). (**I**) Quantitative analysis of tube formation assay. Data were shown as mean ± SEM (* *p* < 0.05; ** *p* < 0.01).

Forkhead box class-O1 (FoxO1) is regarded as an important transcription factor to promote angiogenesis under various condition. In our study, we found that, albeit the expression of total FoxO1 protein was not significantly different in experimental mice muscle tissues, the expression of phosphorylated FoxO1 was significantly reduced in ischemic muscle tissues and was reversed by treatment with RSV (Figure 2A,B). In in vitro experiment, we used HUVECs to further investigate the function of RSV on endothelial cells for angiogenesis. As shown in Figure 2C,D, the total FoxO1 was not significantly different among six groups, and the expression of phosphorylated FoxO1 was significantly reduced under hypoxia condition compared to that under normoxia condition. Under hypoxia condition, the expression of phosphorylated FoxO1 was significantly increased after treated with 0.2 μM and 1 μM of RSV. Moreover, phosphorylation of FoxO1 is highly dependent on nuclear-cytoplasmic shuttling. Therefore, we isolated the cytoplasmic and nuclear protein, respectively, and found that nuclear translocation of FoxO1 was significantly reduced in hypoxia condition following RSV treatment (Figure 2E–G). Then, we performed tube formation assay and found that the length of tube was significantly reduced under hypoxia condition comparing to that under normoxia condition. After 8 h RSV treatment in two different concentrations, the length of tube was longer than that in the DMSO group under hypoxia condition (Figure 2H,I). Taken together, these results indicated that RSV increased phosphorylated FoxO1 and promoted angiogenesis in HUVECs in an ischemic condition.

### 2.3. RSV Promotes Angiogenesis in a FoxO1-Dependent Manner

**Figure 3:**
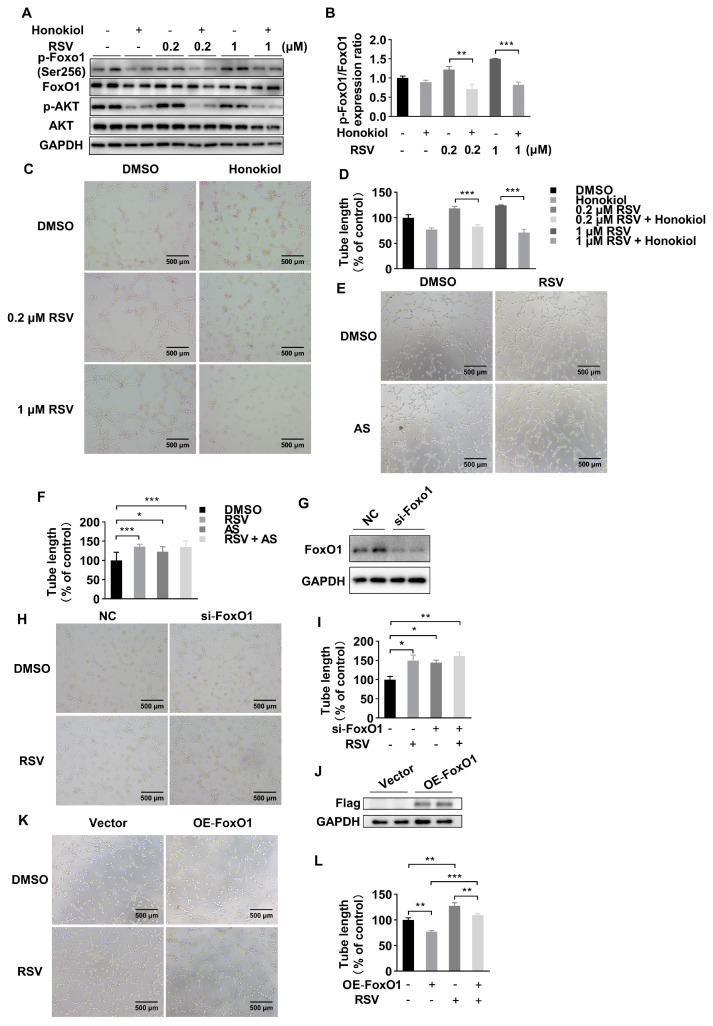
RSV improved the angiogenesis of HUVECs in a FoxO1-dependent manner. (**A**) The expression of phosphorylated FoxO1, FoxO1, phosphorylated Akt and Akt after RSV or/and honokiol treatment. (**B**) Quantitative analysis of Western blot. Data were shown as mean ± SEM (** *p* < 0.01; *** *p* < 0.001). (**C**) The tube formation assay was performed in HUVECs treated with DMSO, 10 μM honokiol, 0.2 μM RSV, 0.2 μM RSV + 10 μM honokiol, 1 μM RSV or 1 μM RSV + 10 μM honokiol for 12 h in a hypoxia condition (100×, scale bar = 500 μm). (**D**) Quantitative analysis of tube formation assay. Data were shown as mean ± SEM (*** *p* < 0.001). (**E**) The tube formation assay was performed in HUVECs treated with DMSO, 0.1 μM AS, 1 μM RSV or 0.1 μM AS + 1 μM RSV for 8 h in a hypoxia condition (100×, scale bar = 500 μm). (**F**) Quantitative analysis of tube formation assay. Data were shown as mean ± SEM (* *p* < 0.05, *** *p* < 0.001). (**G**) The effect of siRNA transfected HUVECs was verified by Western blot. (**H**) The tube formation assay was performed in transfected HUVECs treated with DMSO or 1 μM RSV (100×, scale bar = 500 μm). (**I**) Quantitative analysis of tube formation assay. Data were shown as mean ± SEM (* *p* < 0.05; ** *p* < 0.01). FoxO1-Flag or empty plasmid was transfected in HUVECs, and the overexpression of FoxO1 was determined by Western blot (**J**). (**K**) The tube formation assay was performed in transfected HUVECs treated with DMSO or 1 μM RSV (100×, scale bar = 500 μm). (**L**) Quantitative analysis of tube formation assay. Data were shown as mean ± SEM (** *p* < 0.01; *** *p* < 0.001).

The phosphorylated FoxO1 is regulated by PI3K-Akt pathway. To further explore whether the protective effect of RSV was FoxO1-dependent under hypoxia condition, we first used honokiol, an Akt-pathway inhibitor, to investigate whether the effect of RSV on phosphorylation of FoxO1 was changed when the Akt-pathway was inhibited. The result demonstrated that honokiol treatment could significantly blunt the elevated expression of phosphorylated FoxO1 induced by the treatment with RSV (Figure 3A,B). In the tube formation assay, honokiol could significantly decrease the tube length in RSV-treated groups (Figure 3C,D). Then we used AS1842856 (AS), a specific FoxO1 inhibitor, to further identify the role of FoxO1 in angiogenesis. As shown in Figure 3E,F, the length of tube formation was significantly elevated in AS pretreated HUVECs. The effect of AS on tube formation was similar with RSV. Moreover, after being treated with both RSV and AS, the length of tube formation was significantly increased compared to the DMSO group but not significantly different compared to the AS or RSV group. Then we used siRNA to knock down the expression of FoxO1. The knockdown efficiency was presented by Western blot (Figure 3G). The tube formation assay indicated that the tube length was significantly increased in si-FoxO1 treated HUVECs compared to that in the NC group. However, RSV could not further increase tube length in si-FoxO1 treated HUVECs (Figure 3H,I). Furthermore, we overexpressed FoxO1 in HUVECs by plasmid transfection. The transfection efficiency was verified by Western blot (Figure 3J). As shown in Figure 3K,L, overexpression of FoxO1 in HUVECs decreased tube formation and the effect was reversed by co-treatment with RSV. Taken together, these data indicated that RSV increased angiogenesis via a FoxO1-dependent manner.

### 2.4. Differential Metabolites Analysis of Skeletal Muscle Samples

**Figure 4:**
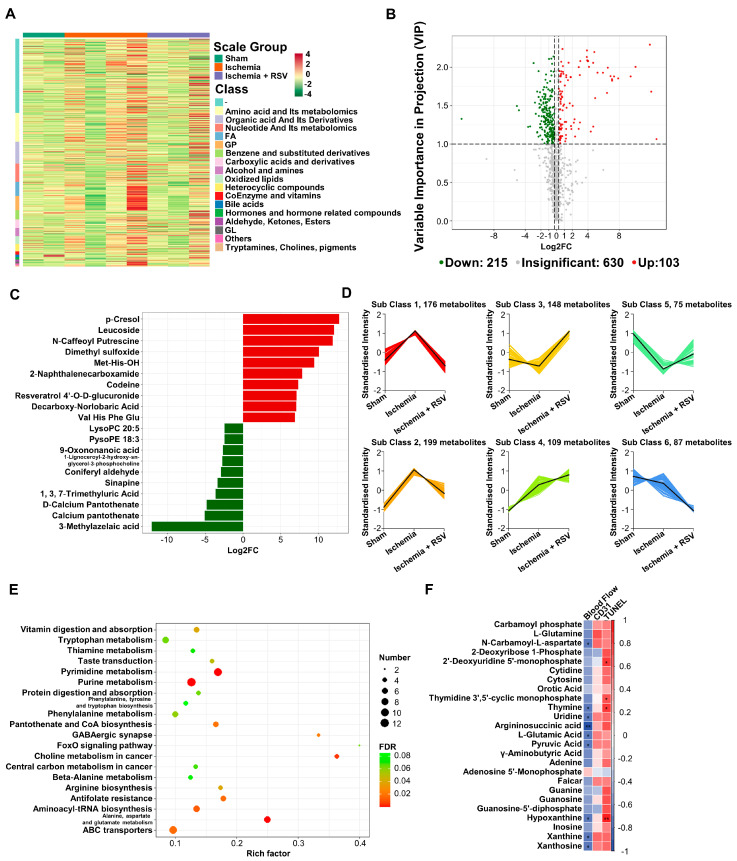
The metabolomic analysis of the gastrocnemius muscles. (**A**) Heat map was performed to show the metabolites of all experimental groups. The row clustering tree represents a metabolite clustering and each column represents sample replication. Normalized signal intensities of metabolites (unit variance scaling) are visualized as color spectrum: red and green represent high and low level of metabolites, respectively. (**B**) Volcano plot showed the differentially expressed metabolites of the ischemia + RSV group against the ischemia group. Red dots indicate up-regulated metabolites and green dots indicate down-regulated metabolites (Fold change (FC) > 1.2, VIP > 1). (**C**) Bar plot displayed the top 10 up-regulated and down-regulated metabolites (FC > 1.2). (**D**) The K-means was conducted to cluster the metabolites into 6 cluster groups. *X*-axis represents the experimental groups and *Y*-axis represents the relative level of metabolites. (**E**) KEGG enrichment pathway analysis showed the metabolic pathway enriched by the screened metabolites. The bubble size represents the differential metabolites number in each pathway. The bubble color shows the FDR value of each pathway. The bubble graph showed the top 20 pathways with FDR < 0.1. (**F**) Correlation analysis was performed between ischemic muscle metabolites in pyrimidine metabolism, purine metabolism as well as alanine, aspartate and glutamate metabolism pathway and the AUC of blood flow perfusion, CD31 immunohistochemical staining as well as TUNEL quantitative data. The correlation was analyzed using Spearman’s correlation analysis. Cells marked with asterisk depict significance following multiple comparisons. The color scale indicates the level of correlation: red, positive correlation; blue, negative correlation. * *p* < 0.05; ** *p* < 0.01; *n* = 2 (sham group); *n* = 4 (ischemia group); *n* = 3 (ischemia + RSV group).

RSV could regulate various metabolic activities in the body. To delineate whether RSV could change metabolic pathway in ischemic hind limbs, the metabolites from ischemic gastrocnemius muscles in experimental mice were subjected to liquid chromatography-quadrupole time of flight-tandem mass spectrometry (LC-QTOF-MS/MS) analysis. First, we displayed all metabolites detected by heat map (Figure 4A), which suggested the possible functional changes in three groups. We applied the volcano plots to gather comprehensive information on differentially expressed metabolites in muscles of the ischemia + RSV group against the ischemia group. The result indicated that the amount of 215 metabolites were significantly down-regulated while 103 were significantly up-regulated between the two groups (Figure 4B). We displayed top 10 up-regulated and down-regulated metabolites by bar plot (Figure 4C). The result showed that Resveratrol 4’-O-D-glucuronide was significantly increased in the ischemia + RSV group. It was reported that RSV has poor bioavailability, and that it could be metabolized to the glucuronidated and sulfated metabolites in the intestine and liver and subsequently accumulated in different organs [20,21]. In addition to the glucuronide derivatives of RSV, other metabolites such as Sinapine and N-Caffeoyl Putrescine were significantly changed after treatment with RSV in ischemic muscle. The mechanism of changes of these metabolites will be further investigated in the future. Furthermore, we conducted k-means clustering analysis to process all metabolites. As shown in Figure 4D, the metabolites in sub class 1, 2 and 5 were different between the sham group and the ischemia group, and returned to the levels of the sham group after treatment with RSV. Using these screened metabolites, we performed Kyoto Encyclopedia of Genes and Genomes (KEGG) enrichment analysis and applied bubble plot to demonstrate the possible pathway changed among three groups (Figure 4E). The result indicated that the FoxO signaling pathway was enriched (Figure 4E), which further verified that the effect of RSV on ischemic skeletal muscles is in a FoxO1 dependent manner. We also discovered that pyrimidine metabolism, purine metabolism as well as alanine, aspartate and glutamate metabolism were enriched with FDR value < 0.001. To verify whether these metabolic pathways play significant roles in the effect of RSV treatment on hind limb ischemia, we established a correlation study between the differentially expressed metabolites, from three metabolic pathways, and the area under the curve (AUC) of blood flow perfusion data, CD31 histochemical staining as well as TUNEL quantitative value (Figure 4F). The Spearman’s correlation analysis illustrated that many metabolites had a significant correlation with the AUC of blood perfusion and TUNEL quantitative value. For instance, argininosuccinic acid was negatively related to the AUC of blood perfusion value, and hypoxanthine was also negatively related to the AUC of blood perfusion value and positively related to the TUNEL quantitative value, which suggested that RSV might ameliorate hind limb ischemia through these metabolic pathways. The exact mechanism of the metabolic pathway under the treatment with RSV will be the focus of our future research.

## 3. Discussion

PAD has become a serious public health issue globally. It is recognized as the third risk factor which causes atherosclerotic cardiovascular morbidity following coronary artery disease and cerebrovascular disease [2]. CLI is the most severe form of PAD. However, current pharmacotherapy for restoration of blood supply in ischemic tissue is limited. Hence, it is essential to develop new therapy to improve collateral vessels formation and tissue repair in CLI patients. In our study, we found the potential protective effect of RSV on CLI.

Firstly, we identified that RSV could improve hind limb ischemia through pro-angiogenesis. In previous studies, it remains controversial regarding the role of RSV in angiogenesis. Wen et al. identified a protective role of RSV in diabetic nephropathy through inhibiting VEGF-mediated angiogenesis [22]. Similarly, Hu et al. found that RSV could bind to VEGF to suppress neovascularization in HUVECs [23]. In contrast, Liu et al. found that RSV pretreatment of bone marrow-derived mesenchymal stem cells (BMSCs) had a better therapeutic efficacy in severe acute pancreatitis due to the promotion of angiogenesis and inhibition of apoptosis [24]. Moreover, Simão et al. reported that RSV has proangiogenic effects on brain endothelial cells to protect the brain after a stroke [25]. Since the microenvironment such as blood glucose and oxygen concentrations varied in cells and tissues and the drug concentrations were different, RSV may have distinct effects on angiogenesis in different pathological states. To clarify the exact effect of RSV on hind limb ischemia, our study observed that the blood perfusion was significantly restored, apoptotic cells were significantly reduced and neovascularization was significantly increased in ischemic muscle tissues after treatment with RSV.

Furthermore, it was reported that the protective effect of RSV is achieved by inhibiting the expression of FoxO1 to promote angiogenesis in hyperglycemia [16]. FoxO1 is regarded as a transcription factor regulating various cellular functions such as oxidative stress, cell longevity and metabolic activity. Roudier et al. found that FoxO1 in endothelial cells acted as a critical regulator for anti-angiogenesis in ischemic muscle [26]. FoxO1 activity is controlled by PI3K-Akt pathway through Akt-mediated phosphorylation. Therefore, the activity or quantity changes of FoxO1 in endothelial cells may play a significant role for angiogenesis switch. Surprisingly, our study demonstrated that phosphorylation of FoxO1 was increased in RSV-treated ischemic skeletal muscles and HUVECs under hypoxia condition. Previous study has demonstrated that phosphorylation of FoxO1 is highly dependent on nuclear-cytoplasmic shuttling [27]. In HUVECs, we also observed a nuclear-cytoplasmic shuttling of FoxO1. It was demonstrated that when HUVECs were treated with RSV in different concentrations in hypoxia circumstance, nuclear localization of FoxO1 was decreased. We performed tube formation assays and the results demonstrated that RSV could increase tube formation. We also used honokiol to inhibit the Akt pathway. The result illustrated that honokiol could reduce the expression of phosphorylated FoxO1 and tube formation, which was increased by RSV. Then the FoxO1 inhibitor (AS) was used to further demonstrate that when FoxO1 was blocked, the proangiogenic function would be activated. Moreover, siRNA was used to knock down the expression of FoxO1, which significantly increased the angiogenesis effect. However, RSV did not increase the tube length in si-FoxO1 treated cells. In contrast, HUVECs with overexpressed FoxO1 led to less tube formation and RSV could reverse the adverse effects of FoxO1 overexpression. Taken together, our results demonstrated that RSV could increase the Akt-mediated phosphorylation of FoxO1 to inhibit nuclear translocation of FoxO1, which promoted angiogenesis.

Metabolomics is a relatively new method of omics that focuses on the metabolites produced by cells. Zhu et al. screened 20 metabolites related to angiogenesis by analyzing the plasma samples of hind limb ischemia model and found a potential protective role of chitosan oligosaccharides in PAD [28]. A previous study has shown that RSV could improve the metabolic activity in many diseases [29]. FoxO1 is regarded as a transcription factor involved in regulating a wide variety of physiological processes, including glucose metabolism, lipogenesis and apoptosis. To further study the changes of metabolism in ischemic muscle after treatment with RSV and to screen out some potentially affected metabolic pathway unbiasedly, in this study, using LC-QTOF-MS/MS analysis on gastrocnemius muscles in mice of experimental groups, we identified that 648 metabolites were different between sham group and ischemia groups, as well as 318 metabolites were significantly different between the ischemia group and the ischemia + RSV group. From the differential expressed metabolites between the ischemia group and the ischemia + RSV group, we found that Resveratrol 4’-O-D-glucuronide was significantly increased in ischemic muscle treated with RSV. Albeit RSV has various pharmacological actions, the major reason for the limited application of this compound is its low bioavailability (<1%). Extensive metabolism of RSV is responsible for the low bioavailability [30]. Previous studies have reported that glucuronide and sulfate derivatives might be produced in the liver or gut and accumulated in different organs including skeletal muscles [31,32]. However, it exhibited weak or even no effect on different cells or disease models [32]. The exact role of this metabolite in ischemic muscles remains to be investigated in the future. Then, we performed k-means clustering analysis and KEGG enrichment analysis to screen the potential metabolic pathways changed among three groups. Surprisingly, pathway enrichment analysis of these metabolites demonstrated that FoxO signaling pathway activity was altered in the condition of ischemia and after treatment with RSV. In addition to FoxO signaling pathway, pyrimidine metabolism, purine metabolism, as well as alanine, aspartate and glutamate metabolism, were enriched with FDR value <0.001. It is reported that these three metabolic pathways would be disrupted in ischemic diseases [33,34,35,36], which indicated that RSV might have regulatory effects on these pathways to protect ischemic hind limbs. Finally, we performed a correlation between the differential metabolites and the AUC of blood flow perfusion data, CD31 histochemical staining and the TUNEL quantitative value. Many metabolites have a significant correlation with the phenotypic data, which verify the role of these pathways in the ischemic muscle treated with RSV. Many previous studies had illustrated that differential metabolites, such as hypoxanthine, inosine, L-glutamine, cytidine, thymine and uridine, matching to these pathways, were changed under ischemic condition [34,35]. Some of these, such as hypoxanthine, was regarded as a marker of ischemia [34], an increase of these metabolites represented a restored level of blood flow in RSV-treated ischemic muscles. Some of the metabolites, such as L-glutamine and uridine, demonstrated a protective effect on hypoxia condition [37,38]. Some metabolites such as L-glutamic acid and argininosuccinic acid have negative effects on the body and accumulate under ischemia condition [39]. After being treated with RSV, they are reduced significantly. However, the mechanism of the effect of RSV on these metabolites and the regulatory relationship between FoxO1 and these pathways remain unclear, and will be our future research direction. Moreover, multiple strategies, i.e., nanocarriers mediation [40] to enhance RSV bioavailability is demanded. However, several limitations exist in our study. The number of experimental animals per group is relatively small in in vivo study. We manipulated FoxO1 in vitro and identified the role of FoxO1 in HUVECs. However, the effect of RSV on FoxO1 knockout or overexpressed hind limb ischemia mouse model remains unclear. Moreover, FoxO1 acts as a transcription factor to regulate cellular function. Additional studies are needed to determine the exact effect of FoxO1 on the downstream pathways in ischemic muscle tissue.

In conclusion, our study indicates that the utilization of RSV could reduce the activity and alter the cellular distribution of FoxO1, which would improve collateral vessels formation and limb ischemia. This investigation potentially provides a novel pharmacotherapeutic treatment strategy for PAD patients.

## 4. Materials and Methods

### 4.1. Drug and Animals

Resveratrol (CAS: 501-36-0) was purchased from MedChemExpress Company (Princeton, NJ, USA, Lot # 58706).

Six-week-old C57BL/6JGpt mice (*n* = 12) weighing 21 ± 2 g were purchased from GemPharmtech Company (Nanjing, China). All animals were maintained in a specific pathogen-free room in cages with individual ventilation at temperature of 23 ± 2 °C and 45 ± 5% humidity with a 12/12 h light-dark cycle and standard food and water feeding ad libitum. The mice were maintained in the laboratory animal center until ten-week-old for the subsequent experiments. The experimental protocols performed on the animals were approved by the Institutional Review Board of The First Affiliated Hospital of Sun Yat-sen University (Guangzhou, China) [permit number, 2018 (35)]. All animal experimental procedures were performed in accordance with the relevant guidelines.

### 4.2. Hind Limb Ischemia Model Establishment and Function Assessment

The 10-week-old mice were divided randomly into three groups: (1) sham surgery group (sham group, *n* = 4); (2) hind limb ischemia group (Ischemia group, *n* = 4); (3) hind limb ischemia + RSV group (ischemia + RSV group, *n* = 4). One week before establishing the hind limb ischemia model, RSV, suspended in 50% saline, 40% PEG400, and 10% DMSO, was administrated 40 mg/kg/day per mouse by gavage each day.

To establish the hind limb ischemia model, mice anaesthetized with isoflurane were placed in the heating mat to maintain body temperature. The left femoral artery was isolated and further ligated on the proximal and distal ends as well as the branches between these two ends. Then the femoral artery was severed and the surgical incision was closed. In the sham group, left femoral artery was isolated with ligation for 5 min and then relieved. After operation, a laser Doppler imager (blood perfusion imager; Perimed AB, Stockholm, Sweden) was used to determine the model success and perfusion ratio as described previously [41]. After surgery, RSV was continued to administrate for a week. During this period, laser Doppler imager was used to serially monitor the blood perfusion recovery of the ischemic hind limbs. After 8 days, the mice were sacrificed, and the gastrocnemius muscles were extracted for further experiments.

### 4.3. Terminal-Deoxynucleotidyl Transferase Mediated Nick End Labeling (TUNEL) Assay

Paraffin embedded gastrocnemius tissues were cut into 4 μm thick sections and mounted on slides. The slides were deparaffinized in xylene, dehydrated in gradient ethanol of 95%, 90%, 80%, and 70% ethanol, respectively, and washed in distilled water. The tissues were marked with liquid blocker pen, incubated in proteinase K working solution at 37 °C for 20 min to retrieve antigen, immersed in 3% H_2_O_2_ at room temperature in a dark place for 20 min and equilibrized at room temperature for 10 min. The mixture of TDT enzyme, dUTP and buffer at 1:5:50 ratio was added to the tissue placed in a flat wet box at 37 °C for 1 h. Then the tissues were incubated with reagent streptavidin-HRP at 37 °C for 30 min and developed the chromogenic reaction by diaminobenzidine (DAB). Finally, the nucleus was counterstained with hematoxylin, the slides were dehydrated and the tissue was mounted with resin mounting medium. The tissue stains were visualized and photographed under an inverted phase contrast microscope (Axio Observer Z1, Zeiss, Germany) at 400× magnification and the images were analyzed by ImageJ software (National Institutes of Health, Bethesda, MD, USA).

### 4.4. Immunohistochemical Staining

The paraffin embedded gastrocnemius tissue sections were deparaffinized, rehydrated, antigen retrieved, and endogenous peroxidase blocked following the protocol described above. To seal the tissue, 3% bovine serum albumin (BSA) was used for 30 min at room temperature. Primary antibody CD31 (cell signaling, 1:100) in PBS was added to the sections that were placed in a wet box at 4 °C overnight. On the following day, the sections were washed by PBS and incubated with secondary antibody (Servicebio, 1:200) at room temperature for 50 min. Then the sections were developed using the chromogenic reaction by DAB, counterstained with hematoxylin stain solution, dehydrated and mounted by neutral gum. The sections were visualized and the images acquired by a microscope (DM 2500B, Leica, Germany) at 200× magnification. The images were analyzed by ImageJ software (National Institutes of Health, Bethesda, MD, USA).

### 4.5. Metabolomics Analysis

Gastrocnemius tissues from three different groups (sham group, *n* = 2; Ischemia group, *n* = 4, ischemia + RSV group, *n* = 3) were homogenized at 30 Hz and methanol with internal standard extract added to obtain sample extracts for on-board analysis. All sample extracts mixed in equal amount were used for LC-QTOF-MS/MS experiment. The metabolites contained in the extracts were identified by combining with the information of multiple databases (Metlin, HMDB, KEGG and MWDB) and quantified by triple quadrupole (QQQ) scans. Then differential metabolites were screened for subsequent analysis.

### 4.6. Cell Culture and Ischemia Treatments

HUVECs (American Type Culture Collection, ATCC, Manassas, VA, USA) were maintained in Kaighn’s modification of Ham’s F-12 medium (F-12K Medium, ATCC, Manassas, VA, USA) supplemented with heparin (Sigma, Burlington, MA, USA) at a final concentration of 0.1 mg/mL, endothelial cell growth supplement (ECGS, combination of Corning™ endothelial cell growth supplement (Fisher Scientific, Waltham, MA, USA) and F-12 K (ATCC, Manassas, VA, USA)) at a final concentration of 0.3 mg/mL and 10% fetal bovine serum (FBS, ATCC, Manassas, VA, USA) at 37 °C with 5% CO_2_.

To investigate the effect of ischemia on cells in vitro, HUVECs were cultured in a hypoxia incubator (2% O_2_, 93% N_2_, 5% CO_2_, Thermo Fisher, Waltham, MA, USA) and F-12K Medium (ATCC, Manassas, VA, USA) supplemented with heparin, ECGS and 2% FBS to simulate ischemic condition *in vitro*. Cells were divided into 6 groups: (1) normoxia control group: cells were incubated in normal condition (95% air and 5% CO_2_); (2) normoxia + 0.2 μM RSV group: cells treated with 0.2 μM RSV were cultured under normal condition for 6 h; (3) normoxia + 1 μM RSV group: cells treated with 1 μM RSV were cultured under normal condition for 6 h; (4) hypoxia control group: cells were cultured under hypoxia (2% O_2_, 93% N_2_, 5% CO_2_, 2% FBS) condition for 6 h; (5) hypoxia + 0.2 μM RSV group: cells treated with 0.2 μM RSV were cultured under hypoxia condition for 6 h; (6) hypoxia + 1 μM RSV group: cells treated with 1 μM RSV were under hypoxia condition for 6 h. To investigate whether the effect of RSV was FoxO1-dependent, we used honokiol (10 μM, MedChemExpress, NJ, USA) as the Akt-pathway inhibitor to prevent the phosphorylation of FoxO1. Cells were divided into 6 groups: (1) control group: cells were treated with DMSO under hypoxia condition for 6 h; (2) Honokiol group: cells were treated with 10 μM honokiol under hypoxia condition for 6 h; (3) 0.2 μM RSV group: cells were treated with 0.2 μM RSV under hypoxia condition for 6 h; (4) 0.2 μM RSV + Honokiol group: cells were treated with 0.2 μM RSV and 10 μM honokiol under hypoxia condition for 6 h; (5) 1 μM RSV group: cells were treated with 1 μM RSV under hypoxia condition for 6 h; (6) 1 μM RSV + Honokiol group: cells were treated with 1 μM RSV and 10 μM honokiol under hypoxia condition for 6 h.

### 4.7. Matrigel Tube Formation Assay

On day one, HUVECs were seeded into a 12-well plate (2 × 10^5^ per well) and divided into 5 groups: (1) normoxia control group: cells were pretreated with DMSO and subjected to normal condition for 8 h; (2) normoxia + 1 μM RSV group: cells were pretreated with 1 μM RSV and subjected to normal condition for 8 h; (3) hypoxia control group: cells were pretreated with DMSO and subjected to hypoxia condition for 8 h; (4) hypoxia + 0.2 μM RSV group: cells were pretreated with 0.2 μM RSV and subjected to hypoxia condition for 8 h; (5) hypoxia + 1 μM RSV group: cells were pretreated with 1 μM RSV and subjected to hypoxia condition for 8 h. Cells treated with honokiol were divided into 6 groups: (1) control group: cells were pretreated with DMSO and subjected to hypoxia condition for 12 h; (2) 0.2 μM RSV group: cells were pretreated with 0.2 μM RSV and subjected to hypoxia condition for 12 h; (3) 1 μM RSV group: cells were pretreated with 1 μM RSV and subjected to hypoxia condition for 12 h; (4) honokiol group: cells were pretreated with DMSO and treated with honokiol under hypoxia condition for 12 h; (5) 0.2 μM RSV + honokiol group: cells were pretreated with 0.2 μM RSV and treated with honokiol and 0.2 μM RSV under hypoxia condition for 12 h; (6) 1 μM RSV + honokiol group: cells were pretreated with 1 μM RSV and treated with honokiol and 1 μM RSV under hypoxia condition for 12 h. Cells treated with AS1842856 were divided into 4 groups: (1) hypoxia + DMSO group: cells were pretreated with DMSO and subjected to hypoxia condition for 8 h; (2) hypoxia + RSV group: cells were pretreated with 1 μM RSV and subjected to hypoxia condition for 8 h; (3) hypoxia + AS group: cells were pretreated with AS1842856 (0.1 μM, MedChemExpress, NJ, USA) and subjected to hypoxia condition for 8 h; (3) hypoxia + RSV + AS group: cells were pretreated with 1 μM RSV and AS and subjected to hypoxia condition for 8 h.

On day two, 50 µL Matrigel matrix (BD Biosciences, San Jose, CA, USA) was coated in precooling 96-well plates. Cells collected from each group were seeded on the Matrigel matrix precoated wells at a density of 2.5 × 10^4^ per well with drugs treating respectively for 4 h at 37 °C in 2% O_2_ condition. Tube formation was inspected and the photographs were taken under an inverted phase contrast microscope (CKX53, Olympus, Tokyo, Japan) at 100× magnification. The length of the tube was measured by ImageJ software (National Institutes of Health, Bethesda, MD, USA). An average of 3 visual fields were selected randomly from each well and each group had at least three duplicates.

### 4.8. Cell Transfection

HUVECs were seeded into a 12-well plate (1 × 10^5^ per well) and plasmid was transfected with FoxO1 overexpressed and vector using Lipofectamine 2000 (Invitrogen, Waltham, MA, USA) on the next day. The mixture of plasmid and Lipofectamine 2000 diluted in Opti-MEM was added into cells with fresh complete medium. The cells were harvested 48 h after transfection for Western blotting and tube formation assay.

For siRNA transfection, HUVECs were transfected with scrambled siRNA (NC) and FoxO1-targeting siRNA (si-FoxO1) using Lipofectamine 2000 according to the manufacturer’s protocol. The final concentration of each siRNA was 100 nM. After transfection for 24 h, the cells were harvested for Western blotting and tube formation assay.

### 4.9. Western Blot

Total proteins were extracted from mice gastrocnemius muscles and HUVECs using radioimmunoprecipitation (RIPA) buffer (Leagene Biotech, Beijing, China). Nuclear and cytoplasm proteins were extracted from HUVECs in lysis buffer (BestBio, Shanghai, China) following instructions from the manufacturer. Proteins were separated by 10% sodium dodecyl sulfate-polyacrylamide gel electrophoresis (SDS-PAGE) and transferred to polyvinylidene difluoride (PVDF) membranes. After blocking with 5% non-fat milk, the membranes were incubated with primary antibodies against FoxO1 (proteintech, 1:1000), Phospho-FoxO1-S256 (ABclonal, 1:1000), Akt (cell signaling, 1:1000), Phospho-Akt-S473 (cell signaling, 1:1000), β-actin (cell signaling, 1:1000), GAPDH (Unibo, 1:4000) and Lamin B1 (cell signaling, 1:1000) respectively at 4 °C overnight. On the following day, the membranes were washed and incubated with HRP-conjugated secondary antibodies (anti-rabbit secondary antibodies, cell signaling, 1:2000; anti-mouse secondary antibodies, cell signaling, 1:1000) at 4 °C for an hour. The protein-antibody interaction bands were detected by enhanced chemiluminescent (ECL) reagent (Millipore, Billerica, MA, USA) and visualized by a chemiluminescence detection system (Amersham Imager 600, GE Healthcare Bio-Sciences AB, Uppsala, Sweden).

### 4.10. Statistics

All results were processed with SPSS software (version 25; Chicago, IL, USA). Data are expressed as the mean ± standard error of mean (SEM). Means of two groups were compared using two-tailed student’s *t*-tests. Comparisons among multiple groups were performed using analysis of variance (ANOVA) followed by Tukey’s multiple-comparison test. A statistically significant difference was considered when the *p* was < 0.05.

For metabolomics analysis, significantly regulated metabolites between groups were determined by VIP ≥ 1 and FC > 1.2. VIP values were calculated by orthogonal partial least squares discriminant analysis (OPLS-DA). KEGG enriched pathways were identified with a hypergeometric test’s *p*-value for the differential expressed metabolites. Correlation test was conducted by Spearman’s correlation analysis. A statistically significant difference was considered when *p* < 0.05.

## Data Availability

All data generated or analyzed during the present study are included in this published article.

## Sample Availability

RSV is available from MedChemExpress (Princeton, NJ, USA).
